# One laboratory’s progress toward accreditation in Tanzania

**DOI:** 10.4102/ajlm.v3i2.202

**Published:** 2014-11-03

**Authors:** Linda R. Andiric, Charles G. Massambu

**Affiliations:** 1 GlobalHealth, American Society for Clinical Pathology (ASCP), Chicago, United States; 2 Ministry of Health and Social Welfare, Tanzania

## Abstract

**Introduction:**

The Amana Regional Hospital Laboratory in Tanzania was selected, along with 11 other regional and district laboratories, to participate in a pilot programme for laboratory quality improvement using the Strengthening Laboratory Management Toward Accreditation (SLMTA) training programme.

**Programme implementation:**

The SLMTA programme entailed hands-on learning, improvement projects between and after a three-workshop series, supervisory visits from an oversight team and an expert laboratory mentor to facilitate and coach the process. Audits were conducted at baseline, exit (approximately one year after baseline) and follow-up (seven months after exit) using the Stepwise Laboratory Quality Improvement Process Towards Accreditation (SLIPTA) checklist. Quality stars (zero to five) were awarded based on audit scores.

**Results:**

With a dedicated staff and strong leadership from laboratory management, Amana Laboratory implemented processes, policies and procedures recommended as elements of best laboratory practices. The laboratory improved from zero stars (36%) at baseline to successfully achieving three stars (81%) at exit. This was the highest score achieved by the 12 laboratories in the programme (the median exit score amongst the other laboratories was 58%). Seven months after completion of the programme, the laboratory regressed to one star (62%).

**Discussion:**

As the SLMTA improvement programme progressed, Amana Laboratory’s positive attitude and hard work prevailed. With the assistance of a mentor and the support of the facility’s management a strong foundation of good practices was established. Although not all improvements were maintained after the conclusion of the programme and the laboratory dropped to a one-star rating, the laboratory remained at a higher level than most laboratories in the programme.

## Introduction

Until recently, laboratories in Africa were largely ignored, distrusted and negated so that, for example, fewer than 50% of patients treated for malaria actually had laboratory confirmation of the disease.^1^ Given the fact that in the United States as many as 94% of all patients’ treatment and diagnoses are based on laboratory data for confirmation,^2^ laboratory utilisation in Africa was, by comparison, undervalued, underused and limited in its capacity. Clinicians often rationalised that laboratory tests were an unnecessary additional cost because diagnoses and treatment protocols were based solely on the physician’s clinical judgement. If or when laboratory data were available, they were perceived as unreliable. If test results were received and were contradictory to clinical impressions, those test results were frequently ignored and only the clinical indicators were considered.^3^

Financial resources from funding organisations and programmes such as the US President’s Emergency Plan for AIDS Relief (PEPFAR) focused on the prevention and care of infectious diseases such as HIV, malaria and tuberculosis.^4^ Because control of these infectious diseases is dependent upon accurate laboratory data, laboratory improvement became a priority. In July 2009 in Kigali, Rwanda, the World Health Organization’s Regional Office for Africa (WHO AFRO), in partnership with the US Centers for Disease Control and Prevention (CDC), the Clinton Health Access Initiative and the American Society for Clinical Pathology (ASCP), launched the Strengthening Laboratory Management Toward Accreditation (SLMTA) training programme,^5^ along with a stepwise accreditation preparation scheme that provides a five-step assessment method rather than a pass-fail one.^6^

In July 2010, the Ministry of Health and Social Welfare (MOHSW) of Tanzania began a pilot programme for the improvement of the country’s medical laboratory services using the SLMTA programme. Twelve hospitals, comprising six regional and six district laboratories, were enrolled in SLMTA as Tanzania’s first cohort. The minimum criteria for selection into the programme, as set by the country’s Laboratory Task Force, were sufficient and qualified staff, a recently remodelled infrastructure to include sufficient space and utilities to carry out quality testing, and a basic knowledge of quality management by prior attendance in a Quality Management Systems (QMS) workshop. Participation in an External Quality Assessment (EQA) programme was also preferred.

This article describes Amana Regional Hospital Laboratory’s success in the improvement of its practices using the SLMTA programme and examines possible factors contributing to this success.

## Programme implementation

Amana Regional Hospital is located in Dar es Salaam City Centre, Tanzania. In 2010, the newly-remodelled hospital laboratory was both spacious and well-endowed with modern laboratory equipment. There were 23 staff members, including phlebotomists and cleaners. The laboratory was maintained neatly and was well organised. When the Tanzanian MOHSW notified Amana Laboratory that it had been selected to participate in the SLMTA programme, the laboratory manager was aware of International Organization for Standardization (ISO) 15189, but he and his staff had no previous knowledge of SLMTA or the WHO AFRO accreditation preparation scheme.

The improvement programme for Amana Laboratory and the other 11 selected facilities began in July 2010 with a baseline audit by three teams of auditors. Two team members were trained South African National Accreditation System (SANAS) auditors, two were ASCP SLMTA facilitators (one of whom was also a College of American Pathologists [CAP] inspector) and three others were SLMTA facilitators from the CDC office in Tanzania. The audits were conducted using the WHO AFRO Stepwise Laboratory Quality Improvement Process Towards Accreditation (SLIPTA) checklist, which is based on ISO 15189. With this checklist, recognition is given for progressive improvement; the lowest level of recognition (one star) requires a minimum of 55% of the total score possible on the checklist, two stars require 65% – 74%, three stars require 75% – 84%, four stars require 85% – 94% and five stars require ≥ 95% compliance. At the highest level (five stars), a laboratory is considered ready to seek accreditation to ISO 15189 standard by any international accreditation agency.^6^

Following the baseline audits, the SLMTA programme consisted of three five-day workshops where participants (laboratory managers and quality officers of the selected facilities) were taught the skills they needed in order to improve the quality of their laboratory services. Each participant received a Laboratory Management Framework document^5^ that defined the tasks laboratory managers must perform in order to meet the standards for best laboratory practices as set forth by the ISO 15189 standard. A copy of the SLIPTA checklist and a SLMTA Toolkit with modules defining best laboratory practices were also given.

The three workshops were held with three-month intervals between them so that skills taught in the workshop could be applied in the participants’ home laboratories. At the conclusion of each workshop, improvement projects were assigned that had either been taught during the previous workshop or were selected to address the common nonconformities found at the baseline audits.

After completion of the programme in August 2011, exit audits were conducted in the 12 laboratories by the same team that conducted the baseline audits. In February 2012, seven months later, an official WHO AFRO SLIPTA audit was conducted in Amana Laboratory by MOHSW on behalf of the African Society for Laboratory Medicine.

## Results

**FIGURE 1:**
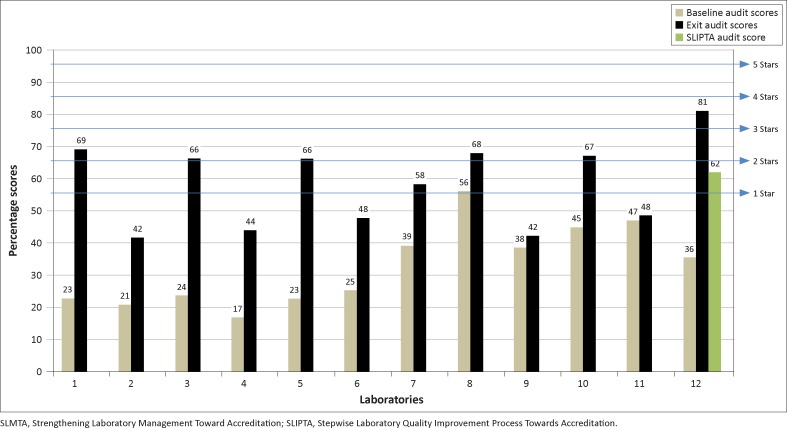
Baseline and exit audit scores for the 12 laboratories in the first SLMTA cohort in Tanzania, including SLIPTA audit score for Laboratory 12, the Amana Laboratory.

The median baseline score for the 12 laboratories was 31% (Figure 1). Amana Laboratory scored 36% at the baseline audit, well below the 55% needed for a one-star rating. During the audit debrief, it was clear that this low score was both a surprise and a disappointment to the Amana laboratory staff. The laboratory manager, quality officer and mentor, an experienced Tanzanian laboratory professional with good laboratory practices assigned to assist Amana Laboratory in its implementation of the improvement projects, responded positively, however, with determination to resolve the underlying quality problems in the laboratory.

By the exit audit, improvements were seen in all of the participating laboratories, with a median exit score of 62% (Figure 1). Amana Laboratory earned a score of 81% at the exit audit, corresponding to a three-star recognition level, which was the highest score in the cohort. Much had been accomplished. For example, a quality manual for the laboratory had been developed by the quality officer in collaboration with the laboratory manager, which documented policies on how the laboratory would ensure quality in every aspect of service delivery. Each policy was either described in detail in the quality manual or was referenced to a full standard operating procedure (SOP) denoted by both the document title and control number. However, despite this exemplary improvement, seven months later in the official WHO AFRO SLIPTA audit, the laboratory had regressed to one star (62%).

## Discussion

At the exit audit, all 12 laboratories in Tanzania’s first SLMTA cohort showed improvements in SLIPTA scores, with five sites achieving a two-star recognition level, one achieving one-star recognition and five achieving no stars but showing improvement. Amana Laboratory achieved three stars and the highest score in the cohort, standing out as an example of the remarkable progress that can be achieved within a single year.

When asked about the important factors that contributed to the successful outcome, the laboratory manager responded that the programme, in its entirety, contained all the essentials that were important for success. Firstly, the tools and job aids taught and practised in the SLMTA programme, including the Laboratory Management Framework and audit checklist, provided the crucial ‘how-to’ guidance for accomplishing best laboratory practices. Secondly, there were supportive visits by the Tanzanian SLMTA supervisory team that provided guidance for the implementation of improvement projects and clarified some translation misunderstandings that resulted from the workshops being taught in English rather than the native Kiswahili. Thirdly, encouragement from the facility medical officer and the regional medical officer was important with regard to supporting the efforts of the laboratory as they implemented improvements. Fourthly, the assigned laboratory mentor assisted in all aspects of implementing the improvements and reinforced the skills learned from the SLMTA curriculum. Finally, the laboratory staff’s team spirit, involvement and commitment to achieveing quality were crucial in meeting the goals of the programme.

Whilst all 12 laboratories in the cohort underwent the same training and supportive supervisory visits, it is possible that varied levels of support from facility medical officers contributed to differences in outcomes. In addition, Amana Laboratory’s relatively greater improvement could be attributed to laboratory leadership; Amana’s laboratory manager made an effort to inspire a team spirit within his staff which, in turn, may have motivated them to strive for achievement of the improvement goals. Also important was maintaining an open mind and positive attitude, despite the low baseline audit score, as Amana’s laboratory management team approached the SLMTA improvement projects. They took the SLMTA curriculum seriously and were diligent in its implementation. They believed in the overall goal and the value of this initiative for the improvement of laboratory service and, by example to their staff, dedicated themselves to the extra hours and hard work required. The establishment of these essential elements ensured that the SLMTA curriculum was utilised to its fullest potential; the supervisory team was welcomed; and the mentor was well-received, enabling him to assist and to carry out his assignment.

Despite Amana Laboratory staff’s remarkable success during the programme, they were not able to maintain the improvements after the programme ended. Seven months later, the score had dropped to the one-star level. As is often the case, once the focus on the programme ended, the laboratory found it difficult to maintain and update records and reviews. Amana Laboratory has learned that quality improvement is a continuous process and that, whilst progress may not always be steady, they are committed to continue to both improve and implement their quality systems.
